# Circular RNAs in Breast Cancer: An Update

**DOI:** 10.3390/biom14020158

**Published:** 2024-01-29

**Authors:** Haolin Bao, Jiehan Li, Qihang Zhao, Qingling Yang, Yi Xu

**Affiliations:** 1 Department of Hepatopancreatobiliary Surgery, The Second Affiliated Hospital of Harbin Medical University, Harbin 150086, China; 2 Department of Mammary Surgery, The Second Affiliated Hospital of Harbin Medical University, Harbin 150086, China; 3 Anhui Province Key Laboratory of Cancer Translational Medicine, Bengbu Medical University, Bengbu 233030, China; 4 State Key Laboratory of Oncology in South China, Cancer Center of Sun Yat-Sen University, Guangzhou 510060, China; 5 Shanghai Key Laboratory of Molecular Imaging, Shanghai University of Medicine and Health Sciences, Shanghai 201318, China; 6 Department of Pathology, Li Ka Shing Faculty of Medicine, The University of Hong Kong, Hong Kong SAR 999077, China; 7 Research of Zhejiang Province, The First Affiliated Hospital of Wenzhou Medical University, Wenzhou 325000, China

**Keywords:** circular RNA, breast cancer, biomarker, treatment, exosome

## Abstract

Breast cancer (BC), characterized by high heterogeneity, is the most commonly reported malignancy among females across the globe. Every year, many BC patients die owing to delayed diagnosis and treatment. Increasing researches have indicated that aberrantly expressed circular RNAs (circRNAs) are implicated in the tumorigenesis and progression of various tumors, including BC. Hence, this article provides a summary of the biogenesis and functions of circRNAs, as well as an examination of how circRNAs regulate the progression of BC. Moreover, circRNAs have aroused incremental attention as potential diagnostic and prognostic biomarkers for BC. Exosomes enriched with circRNAs can be secreted into the tumor microenvironment to mediate intercellular communication, affecting the progression of BC. Detecting the expression levels of exosomal circRNAs may provide reference for BC diagnosis and prognosis prediction. Illuminating insights into the earlier diagnosis and better treatment regimens of BC will be potentially available following elucidation of deeper regulatory mechanisms of circRNAs in this malignancy.

## 1. Introduction

Breast cancer (BC) remains the most frequently diagnosed malignancy and constitutes the second leading cause of cancer-associated deaths in women worldwide [1,2]. Based on the status of ER, PR, Ki-67 and HER2 (ERBB2), BC characterized by significant heterogeneity can be categorized into four major subtypes, namely, luminal A, luminal B, HER2-positive and triple-negative breast cancer (TNBC) [3]. Among these BC subtypes, luminal A and luminal B subtypes are the most common tumor subtypes in early-stage female BC patients with a slower rate of progression and a higher incidence of recrudescence [4,5]. HER2-positive BC is characterized by HER2 overexpression and unfavorable prognosis [6]. TNBC is a highly invasive malignancy with particularly dismal prognosis and, owing to deficiency of specific targets, such as ER, PR and HER2, there are no satisfactory TNBC treatment regimens [7]. Patients with early-stage BC have a 5-year survival rate of over 90%, whereas this rate declines dramatically to approximately 27% once metastasis occurs [8]. Hence, seeking novel molecular markers and therapeutic targets is of utmost significance to ameliorating the survival and prognosis of BC patients. 

CircRNAs, a newly discovered group of endogenous noncoding RNAs (ncRNAs), can be found in diverse tissues and cells [9]. Unlike linear RNAs, such as microRNAs (miRNAs) and long noncoding RNAs (lncRNAs), circRNAs possess covalently closed loop structures lacking 5′-to-3′ polarity and polyadenylated tails, by which they could resist degradation by exonucleases and therefore acquire high stability [10,11]. Several studies have suggested that circRNAs, aberrantly expressed in various malignant tumors, affect tumorigenesis and development through diverse mechanisms. For example, hsa_circRNA_102002 was upregulated in papillary thyroid cancer (PTC) tissues and cells, and depletion of hsa_circRNA_102002 suppressed metastasis of PTC by regulation of the miR-488-3p/HAS2 axis [12]. CircFIRRE was revealed to facilitate the progression and metastasis of osteosarcoma via tumorigenic–angiogenic coupling [13]. In ovarian cancer, circ_0000554 was found to promote cell invasion, proliferation and epithelial–mesenchymal transition (EMT) by acting as a sponge for miR-567. Conversely, the suppression of circ_0000554 resulted in contrary outcomes [14]. Consequently, targeting circRNAs appears to contribute to improving the therapeutic strategies for cancer.

In this review, we aim to recapitulate the existing knowledge of the biogenesis and functions of circRNAs, as well as their involvement in BC occurrence and progression, so as to provide innovative approaches for BC treatment.

## 2. Biogenesis of circRNAs

CircRNAs were initially depicted in RNA viruses in the 1970s and further identified in the cytoplasm of eukaryotic cells using electron microscopy [15,16]. CircRNAs are formed through precursor mRNA (pre-mRNA) back-splicing, which differs from the classical splicing mechanism used for producing linear RNAs [17]. Based on their intrinsic sequences, circRNAs are divided into three diverse categories: exonic circRNAs (EcircRNAs), intronic circRNAs (ciRNAs) and exon–intron circRNAs (EIcircRNAs) [18]. EcircRNAs are generated by spliceosomal back-splicing, a process attaching the downstream 5′ splice site to the upstream 3′ splice site [19]. If introns between exons are reserved, the resultant circRNAs are termed EIcircRNAs [20]. CiRNAs are estimated to result from intronic lariats that are formed during splicing and cannot debranch because of the existence of RNA sequence motifs near the branch site and 5′ splice site [21]. Presently, three representative biogenesis mechanisms are accountable for triggering the formation of circRNAs, including RNA-binding protein (RBP)-mediated cyclization, intron pairing-driven cyclization and lasso-driven cyclization [22]. Some RBPs, such as Quaking (QKI), facilitate circRNA formation through binding to single-stranded RNA (ssRNA) motifs in flanking introns of cyclized exons [23]. In terms of intron pairing-driven cyclization, base-pairing between flanking intronic complementary sequences induces the combination of 5′ splice donor site of pre-mRNA and 3′ splice acceptor site, thus boosting circRNA formation [24,25]. In lasso-driven cyclization, exon skipping event, which occurs during pre-mRNA splicing, causes the formation of exon–intron-containing lasso intermediate that forms a circRNA through back-splicing [26].

## 3. Functions of circRNAs

### 3.1. MiRNA Sponge

MiRNAs, which are a type of ncRNAs, have been extensively studied in eukaryotic cells as post-transcriptional regulators of gene expression. They typically consist of 19–25 nucleotides [27]. Multiple studies have validated the role of circRNAs as miRNA sponges. For example, circHMGCS1-016 was uncovered to reshape immune surroundings in intrahepatic cholangiocarcinoma (ICC) by controlling the levels of CD73 and GAL-8 through interaction with miR-1236-3p [28]. CircDLG1 sponged miR-141-3p and enhanced the expression of CXCL12, which in turn facilitated the progression of gastric cancer and contributed to resistance against anti-PD-1 treatment [29]. Hsa_circ_0014130 was found to exert oncogenic effects in bladder cancer by upregulation of KCNJ12 expression via acting as a miR-132-3p sponge [30]. CircZNF236 was observed to enhance oral squamous cell carcinoma (OSCC) development through sponging miR-145-5p and consequently boosting MBTD1 expression [31].

### 3.2. Interacting with Proteins

A few circRNAs have been observed to perform their functions through interacting with proteins. For instance, by interacting with MEK1, circNFIB (also called hsa_circ_0086376) promoted the dissociation between ERK2 and MEK1, leading to the repression of ERK signaling [32]. CircWSB1, upregulated by HIF1α under hypoxia, can competitively bind to USP10, a deubiquitinase, preventing the access of p53 to USP10, thus resulting in degradation of P53 [33]. The interaction between circURI1 and hnRNPM was confirmed to regulate alternative splicing of genes associated with cell migration in gastric cancer [34]. CircDIDO1 (also termed hsa_circ_0061137) was revealed to bind to PRDX2 and facilitate RBX1-mediated PRDX2 ubiquitination and degradation [35]. Binding of circST6GALNAC6 to the N-terminus of small HSPB1 was disclosed to retard the phosphorylation of Ser-15 site of HSPB1 induced by erastin, resulting in activation of p38 MAPK pathway and subsequent enhancement of cell ferroptosis in bladder cancer [36]. The role of circEIF3I (also referred to as hsa_circ_0011385) in promoting PTC progression was demonstrated by its interaction with AUF1, which ultimately facilitated the production of Cyclin D1 protein [37].

### 3.3. Affecting Parental Gene Expression

Studies have demonstrated that several circRNAs participate in regulation of their parental gene expression. For example, one group revealed that circSMARCA5 could bind to its parental gene locus to form an R-loop, causing transcriptional arrest of SMARCA5 exon 15 [38]. Another group demonstrated that SIRT1 expression could be upregulated post-transcriptionally by circ-SIRT1 (hsa_circ_0093884) via sponging miR-5195-3p/miR-3681-3p. Notably, this group also uncovered that circ-SIRT1 contributed to stabilizing SIRT1 protein post-translationally via recruiting USP22 and further accelerating deubiquitination on SIRT1 protein [39]. Additionally, circTTN, a circular intronic RNA, was confirmed to repress the transcription and myogenesis of TTN through forming heterotypic complexes via recruiting PURB proteins [40]. An antisense circRNA, named circSCRIB, was illustrated to retard the splicing and translation of its parental gene SCRIB, enhancing BC progression [41].

### 3.4. Encoding Proteins or Peptides

Although circRNAs were thought to be non-protein-encoding RNAs with no open reading frames (ORFs), they have been discovered to possess the capability of encoding proteins or peptides [42,43]. For instance, a study discovered that circRNA GGNBP2, induced by IL-6, boosted cell growth and metastasis in ICC by encoding a protein, named cGGNBP2-184aa, which bound to STAT3 and phosphorylated STAT3Tyr705 [44]. In addition, a novel protein encoded by circDIDO1, named DIDO1-529aa, was discovered to attenuate the activity of PARP1 by directly interacting with it [35]. Furthermore, one group revealed that circ-EIF6 encoded a peptide, called EIF6-224aa, which reduced MYH9 degradation via suppressing the ubiquitin–proteasome pathway, further activating the Wnt/beta-catenin pathway and accelerating TNBC progression [45]. The interaction between FBXW7 and mTOR, facilitated by circZKSCAN1-encoded circZKSaa, was verified to boost mTOR ubiquitination in hepatocellular carcinoma (HCC), consequently suppressing the PI3K/AKT/mTOR pathway [46]. CircMTHFD2L was elucidated to encode a protein, named CM-248aa, which could induce dephosphorylation of AKT, P65 and extracellular signal-regulated kinase through targeting the SET acidic domain and subsequently suppressing the SET-protein phosphatase 2A interaction [47].

## 4. CircRNAs in Breast Cancer

**Figure 1:**
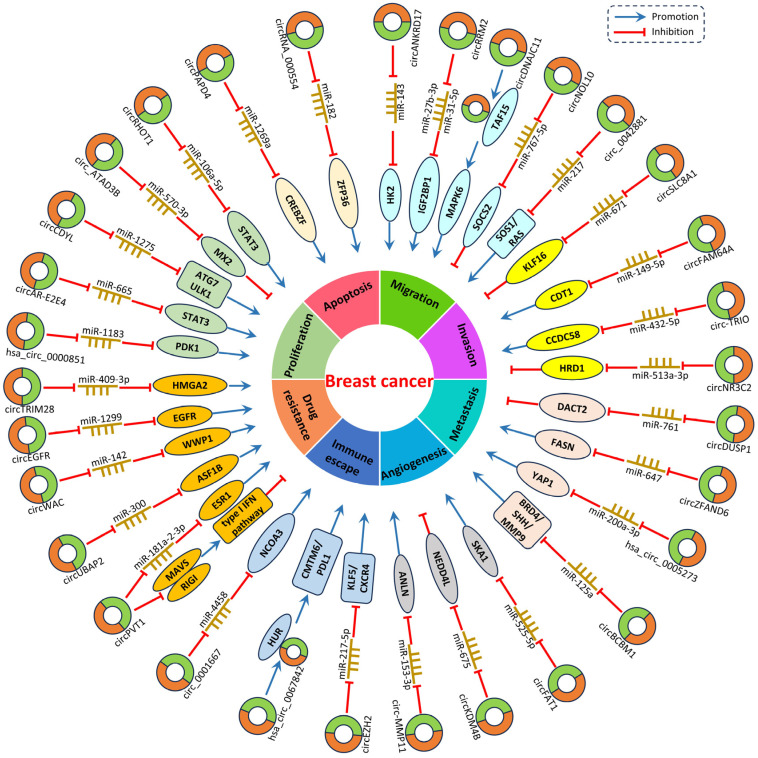
Regulatory mechanisms of circRNAs in BC progression.

Growing evidence suggests that multiple circRNAs exhibit abnormal expression in BC; however, the precise mechanisms through which circRNAs influence the onset and progression of BC remains unclear. Therefore, we discuss the circRNA-associated studies in BC in recent years (Table 1 and Figure 1).

### 4.1. CircRNA Expression Profiles in BC

Aberrant expression of circRNAs in BC has been reported in a significant quantity. Li et al. performed RNA sequencing on BC tissues and matched normal tissues and discovered 148 differentially expressed (DE) circRNAs, of which 70 were upregulated and 78 were downregulated [48]. According to circRNA microarray analysis of six pairs of BC tissues and adjacent normal breast tissues, Li et al. discovered 218 DE circRNAs, including 89 downregulated and 129 upregulated circRNAs [49]. By performing circRNA high-throughput sequencing, Xu et al. identified 17,966 different circRNA candidates (11,375 novel circRNAs and 6591 existing circRNAs) in three pairs of BC and paracancer tissues that exhibited metastasis in the axillary lymph nodes. Afterwards, they identified 136 circRNAs that exhibited significant overexpression in BC tissues when compared to matched paracancer tissues using a paired-samples *t*-test [50]. Zheng et al. conducted circRNA microarray analysis in four pairs of BC tissues and adjacent nontumor tissues and found 256 significantly upregulated circRNAs and 277 significantly downregulated circRNAs. Importantly, the quantitative real-time PCR (qRT-PCR) assay results unveiled that hsa_circ_0001583 showed significant upregulation in BC groups (*p* < 0.05) [51]. Li et al. identified 4370 DE circRNAs in six pairs of BC tissues and corresponding non-cancerous tissues by applying competing endogenous RNA (ceRNA) microarray probes, of which 2375 and 1995 circRNAs were increased and decreased, respectively. Subsequent Gene Ontology (GO) and Kyoto Encyclopedia of Gene and Genome (KEGG) analyses unveiled that these DE circRNAs were related to DNA replication, cell cycle, BC and familial BC [52]. Yuan et al. utilized circRNA microarray to detect 3653 DE circRNAs in ER-positive BC tissues compared to adjacent normal tissues. Among these DE circRNAs, 1700 circRNAs were downregulated and 1953 circRNAs were upregulated [53]. Utilizing high-throughput RNA sequencing, Yuan et al. discovered 16 markedly downregulated and 7 markedly upregulated circRNAs in both the MDA-MB-231 EMT and MCF-7 EMT groups. Additionally, in subsequent study, it was noted that circSCYL2 expression was reduced in BC tissues [54]. To investigate the circRNA expression profile of BC brain metastasis (BCBM), Fu et al. performed RNA-sequencing and identified 191 downregulated and 215 upregulated circRNAs in 231-BR cells relative to MDA-MB-231 cells [55]. Table 2 summarizes the circRNA expression profiles in BC.

**Table 1:** Dysregulated circRNAs in BC.

CircRNAs	Expression	Target miRNAs	Related Genes and Pathways	Biological Functions	Reference
circEZH2	Upregulated	miR-217-5p	KLF5/CXCR4	Promotes proliferation, invasion, migration, EMT, metastasis	[56]
circ_0042881	Upregulated	miR-217	SOS1/RAS	Promotes proliferation, invasion, migration, metastasis	[57]
circAR-E2E4	Upregulated	miR-665	STAT3	Promotes proliferation	[58]
circZFAND6	Upregulated	miR-647	FASN	Promotes proliferation, metastasis	[59]
hsa_circ_0000851	Upregulated	miR-1183	PDK1/p-AKT	Enhances proliferation, migration	[60]
hsa_circ_0067842	Upregulated	/	HuR/CMTM6/PD-L1	Promotes metastasis, invasion, migration, immune escape	[49]
circANKRD17	Upregulated	miR-143	HK2	Promotes growth, invasion, migration, cell cycle progression	[61]
circRRM2	Upregulated	miR-31-5p/miR-27b-3p	IGF2BP1	Promotes invasion, migration, metastasis	[62]
circDNAJC11	Upregulated	/	TAF15/MAPK6	Enhances proliferation, migration, invasion, metastasis	[63]
circFAM64A	Upregulated	miR-149-5p	CDT1	Promotes proliferation, invasion, migration, cell cycle progression	[64]
circCDYL	Upregulated	miR-1275	ATG7, ULK1	Promotes autophagy, proliferation	[65]
circRHOT1	Upregulated	miR-106a-5p	STAT3	Enhances proliferation, invasion, migration. Inhibits apoptosis, ferroptosis	[66]
hsa_circ_0005273	Upregulated	miR-200a-3p	YAP1, Hippo pathway	Promotes proliferation, migration, metastasis. Regulates cell cycle.	[67]
circEGFR	Upregulated	miR-1299	EGFR	Promotes proliferation, invasion, migration, EMT, THP resistance	[68]
circBCBM1	Upregulated	miR-125a	BRD4/SHH/MMP9	Enhances proliferation, migration, metastasis	[69]
circ-TRIO	Upregulated	miR-432-5p	CCDC58	Promotes proliferation, invasion, migration, metastasis	[70]
circKDM4B	Downregulated	miR-675	NEDD4L	Suppresses tumor growth, invasion, migration, angiogenesis, metastasis	[71]
circNR3C2	Downregulated	miR-513a-3p	HRD1	Inhibits proliferation, invasion, migration, metastasis	[72]
circ_ATAD3B	Downregulated	miR-570-3p	MX2	Suppresses proliferation	[73]
circNOL10	Downregulated	miR-767-5p	SOCS2, JAK2/STAT5	Suppresses proliferation, invasion, migration, EMT	[74]
circDUSP1	Downregulated	miR-761	DACT2	Inhibits proliferation, invasion, migration, EMT, metastasis	[75]
circPAPD4	Downregulated	miR-1269a	CREBZF	Suppresses proliferation. Promotes apoptosis	[76]
circSLC8A1	Downregulated	miR-671	KLF16, PTEN/PI3k/Akt	Suppresses proliferation, migration, invasion	[77]
circRNA_000554	Downregulated	miR-182	ZFP36	Induces apoptosis, autophagy. Suppresses EMT, invasion, migration, cell cycle progression.	[78]
cSERPINE2	Upregulated	miR-513a-5p	MALT1/ NF-κB	Enhances proliferation, invasion	[79]
circRHOT1	Upregulated	miR-204-5p	PRMT5	Promotes proliferation, invasion, migration, EMT	[80]
circTBPL1	Upregulated	miR-653-5p	TPBG	Promotes proliferation, migration, invasion, metastasis	[81]
circ_0001142	Upregulated	miR-361-3p	PIK3CB	Promotes proliferation, metastasis	[82]

### 4.2. CircRNAs Regulate the Tumorigenesis and Progression of BC

Numerous studies have demonstrated that circRNAs can function as tumor promoters or tumor suppressors to regulate the biological behaviors of BC, such as proliferation, invasion, migration, metastasis, apoptosis, etc. Here, we will have a discussion about the mechanisms of action by which circRNAs are capable of regulating BC tumorigenesis and progression.

#### 4.2.1. Tumor Promoters

CircEZH2 (hsa_circ_0008324) was shown to be highly expressed in BC cell lines compared with normal breast epithelial cells [56]. Subsequently, upregulated circEZH2 was also found in BC-derived liver metastases (BCLM) specimens compared with BC primary specimens [56]. In addition, Kaplan–Meier results suggested that BC patients with circEZH2 overexpression had a worse prognosis [56]. Moreover, overexpressed circEZH2 remarkably facilitated BC cell proliferation, invasion, migration and metastasis, whereas downregulation of circEZH2 elicited totally opposite outcomes [56]. Furthermore, xenograft nude mice models were established to validate that as compared with the controls, mice with circEZH2 overexpression had more liver nodules, as well as higher luciferase activity [56]. Mechanistically, circEZH2 can adsorb miR-217-5p to elevate the expression of KLF5, which further accelerated EMT of BC via upregulating CXCR4 transcriptionally, thus resulting in BCLM. Interestingly, KLF5 drived the transcription of FUS, which could enhance the back-splicing of circEZH2, forming a novel FUS/circEZH2/KLF5 feedback loop [56]. Circ_0042881, induced by EIF4A3, exhibited increased expression in BC tissues and plasma, and showed strong correlation with clinicopathological features [57]. A CCK-8 assay substantiated that cell proliferation was significantly attenuated after circ_0042881 depletion, whereas circ_0042881 overexpression accelerated cell viability [57]. EdU and colony formation experiments further revealed that depletion of circ_0042881 hindered the proliferation of MDA-MB-231 and MCF-7 cells, whereas circ_0042881 overexpression provoked the phenotypes [57]. A subsequent Transwell assay illustrated that circ_0042881 depletion led to a decrease in the quantity of migrant and invasive cells, while overexpressing circ_0042881 reversed this effect [57]. Furthermore, a divergent alteration in the healing areas following circ_0042881 depletion and overexpression in MCF-7 and MDA-MB-231 cells was observed by wound healing assay [57]. Notably, experimental lung metastasis models elucidated that increased circ_0042881 levels in MDA-MB-231 cells were associated with a higher number of metastatic lung nodules [57]. Mechanistically, circ_0042881 exerted cancer-promoting effects in BC through sponging miR-217 to antagonize its inhibitive effect on SOS1, a guanine nucleotide exchange protein, thus activating RAS protein and initiating downstream signaling cascades, including the PI3K/AKT pathway and MEK/ERK pathway [57]. CircAR-E2E4, derived from exon 2–4 of androgen receptor (AR) gene, was demonstrated to induce the proliferation of TNBC cells (MDA-MB-231 and MDA-MB-468) via CCK-8 assay, while depletion of circAR-E2E4 markedly retarded cell proliferation in MDA-MB-468 and MDA-MB-231 cells [58]. Further research revealed that circAR-E2E4 expedited TNBC cell proliferation through sponging miR-665 to increase the expression of STAT3, a DNA-binding transcription factor, which was determined to be negatively associated with survival rate by overall survival (OS) analysis [58].

CircZFAND6, located at chrX: 80412669-80415142, was demonstrated to be upregulated in BC tissues and cell lines [59]. CCK-8 test demonstrated that the suppression of circZFAND6 significantly inhibited the capacity of BC cells to form colonies. Similarly, utilizing a Transwell assay, Huang et al. confirmed that circZFAND6 downregulation markedly hindered BC cell metastasis [59]. Additionally, inhibition of circZFAND6 reduced lung metastasis in nude mice. Mechanistically, circZFAND6 boosted BC proliferation and metastasis through sponging miR-647 and inducing FASN expression [59]. Notably, EIF4A3 could interact with circZFAND6 pre-mRNA transcript upstream region, achieving circZFAND6 overexpression in BC [59]. Hsa_circ_0000851, derived from the sixth intron of the TCF4 gene, showed overexpression in TNBC cell lines as well as tissue specimens [60]. Subsequent experiments elucidated that upregulated hsa_circ_0000851 interacted with miR-1183 and suppressed its activity, leading to enhanced expression of 3-phosphoinositide-dependent protein kinase-1 (PDK1), thereby accelerating cell proliferation and migration in TNBC via PDK1/p-AKT [60]. Hsa_circ_0067842 expression was elevated in BC tissues and cells, as testified by detection of 126 BC specimens and 59 adjacent normal tissues [49]. In addition, hsa_circ_0067842 depletion dramatically impeded migration and invasion in MDA-MB-468 and MDA-MB-231 cells, whereas hsa_circ_0067842 overexpression substantially accelerated migration and invasion in MCF-7 and BT-549 cells [49]. Intriguingly, hsa_circ_0067842 exerted no effect on BC cell proliferation, which could be demonstrated by CCK-8 assay and colony formation assay. The aforementioned findings unveiled that hsa_circ_0067842 could enhance the metastasis of BC cells in vitro. Moreover, Li et al. co-cultured MCF-7 and MDA-MB-468 cells with PBMCs and found that the proliferation of PBMCs was suppressed and there was a significant decline in the proportion of CD8^+^ T cells [49]. Notably, depletion of hsa_circ_0067842 caused enhanced PBMC proliferation as well as increased proportion of CD8^+^ T cells, whereas overexpressing hsa_circ_0067842 led to the opposite effect, indicating that hsa_circ_0067842 promoted the immune escape of BC [49]. Mechanistically, hsa_circ_0067842 interacted with HuR to promote its translocation into cytoplasm, strengthening CMTM6 stability and inhibiting PD-L1 degradation via affecting its ubiquitination, ultimately facilitating tumor metastasis and immune escape in BC [49].

CircANKRD17, also referred to as hsa_circ_0001417, was verified to be upregulated in BC tissues and cells. BC cell proliferation was suppressed upon knockdown of circANKRD17, as demonstrated by EDU and CCK-8 assays [61]. Additionally, overexpression of circANKRD17 enhanced cell invasion and migration in BC, while circANKRD17 knockdown repressed these phenotypes [61]. Furthermore, subsequent cell cycle analysis illustrated that knockdown of circANKRD17 diminished the cell number in the S phase, whereas overexpression of circANKRD17 markedly facilitated cell cycle progression [61]. Mechanistically, circANKRD17 could act as a miR-143 sponge to overthrow the inhibitive effect of miR-143 on HK2, accelerating glycolysis in BC cells and thus boosting cell growth, invasion, migration and cell cycle progression [61]. CircRRM2 (hsa_circ_0052582) was confirmed to be upregulated in tumor tissues of BC patients, and highly expressed circRRM2 was correlated with advanced N stage in BC patients [62]. Gain and loss of function experiments demonstrated that circRRM2 was capable of promoting BC cell migration and invasion [62]. Mechanism researches revealed that circRRM2 accelerated BC cell migration and invasion through upregulating IGF2BP1 expression via sponging miR-31-5p/miR-27b-3p [62]. Interestingly, IGF2BP1 can interact with MYC, which enforced the transcriptional circRRM2 activation, thereby forming a positive feedback loop and inducing the metastasis in patients with BC [62]. CircDNAJC11, mainly distributed in the cytoplasm, showed overexpression in BC cells, especially in MCF-7 and SK-BR-3 cells [63]. Furthermore, overexpressed circDNAJC11 was also detected in BC tissues through qRT-PCR analysis of circDNAJC11 expression in 80 pairs of BC and neighboring non-tumor tissues [63]. Further studies illustrated that circDNAJC11 facilitated proliferation, invasion, migration and metastasis in BC cells, as well as expedited the growth of xenograft tumors in nude mice [63]. In terms of mechanism, circDNAJC11 accelerated the malignant progression of BC by interacting with TAF15 to augment MAPK6 expression [63]. Upregulated expression of circFAM64A was observed in TNBC tissues and cells, and circFAM64A overexpression portended unfavorable prognosis in patients with BC [64]. In vitro experiments revealed that circFAM64A could enhance TNBC cell proliferation, migration, invasion and accelerate cell cycle progression [64]. Mechanistically, circFAM64A exerted oncogenic role in TNBC through increasing CDT1 expression via acting as a miR-149-5p sponge [64].

A study revealed that circCDYL, a circRNA associated with autophagy, exhibited increased expression in BC tissues. This increased circCDYL expression was correlated with larger tumor size, higher Ki67 index, ER negative status and more lymphatic metastasis [65]. Additionally, increased proportion of living cells were observed after overexpressing circCDYL in MCF-7 and MDA-MB-231 cells by cell viability assays, whereas silencing of circCDYL markedly decreased the proportion of living cells [65]. Likewise, a plate colony formation assay confirmed that circCDYL overexpression accelerated colony formation, whereas downregulation of circCDYL led to the opposite effect, suggesting that circCDYL can boost the proliferation of MCF-7 and MDA-MB-231 cells [65]. Surprisingly, despite circCDYL overexpression stimulated autophagy in MDA-MB-231 cells, the expression of circCDYL remained unaffected by autophagy. However, autophagy inhibitor Bafilomycin A1 antagonized the effect of circCDYL upregulation on proliferation of MCF-7 and MDA-MB-231 cells [65]. In terms of mechanism, circCDYL could act as a sponge for miR-1275 to elevate the expression of ATG7 and ULK1, bolstering the autophagic level of MDA-MB-231 cells, thereby facilitating cell proliferation in BC [65]. CircRHOT1 depletion was confirmed to reduce colony formation, migration and invasion and enhance cell apoptosis in MDA-MB-231 and T47D cells [66]. Tumorigenicity analysis disclosed that circRHOT1 facilitated tumor growth in nude mice implanted with MDA-MB-231 cells. In addition, knocking down circRHOT1 significantly elevated the levels of iron, Fe^2+^ and reactive oxygen species (ROS) in MDA-MB-231 and T47D cells, meaning that circRHOT1 could decrease ferroptosis in BC cells [66]. Mechanistically, circRHOT1 promoted the malignant advancement and reduced ferroptosis in BC through regulation of the miR-106a-5p/STAT3 axis [66]. Hsa_circ_0005273 showed overexpression in BC tissues and MCF-7, MDA-MB-231, SKBR3 and HCC-1937 cells, and had positive correlation with tumor volume, TNM stage, lymphatic metastasis and distant metastasis. Nevertheless, there was no observed correlation between hsa_circ_0005273 expression and patient age [67]. MTT and colony formation assays confirmed that depletion of hsa_circ_0005273 retarded the proliferation of MDA-MB-231, SKBR3 and MCF-7 cells. Western blotting analysis showed a decline in the expression of proliferation marker PCNA following the depletion of hsa_circ_0005273 [67]. In addition, a decrease in cell migration was observed in MDA-MB-231 cells using a Transwell assay [67]. Moreover, flow cytometry analysis elucidated that depletion of hsa_circ_0005273 led to increased percentage of G0/G1 phase cells [67]. Furthermore, hsa_circ_0005273 knockdown diminished tumor size and weight as demonstrated by xenograft tumor assay. In terms of mechanism, hsa_circ_0005273 boosted YAP1 expression and deactivated Hippo pathway by adsorbing miR-200a-3p, ultimately facilitating BC progression [67].

CircEGFR (hsa_circ_0080220), derived from exons 2-4 of EGFR gene on chromosome 7p11.2, was disclosed to be overexpressed in TNBC tissues, as well as MDA-MB-468, MDA-MB-231 and HCC-1806 TNBC cells [68]. CCK-8 assay results indicated that circEGFR overexpression remarkably boosted the proliferation of MDA-MB-231 cells, whereas circEGFR knockdown had the opposite impact on MDA-MB-231 cells [68]. The results of EdU assay confirmed that the ratio of EdU-positive cells was increased by circEGFR overexpression and decreased by circEGFR knockdown [68]. Additionally, Transwell and wound healing assays demonstrated that upregulation of circEGFR led to bolstered migratory and invasive abilities in MDA-MB-231 cells, while circEGFR knockdown reduced migration and invasion of MDA-MB-468 cells. Moreover, circEGFR overexpression was elucidated to attenuate E-cadherin expression but enhance vimentin and snail expression in TNBC cells using Western blot analysis, whereas circEGFR knockdown reversed these functions, suggesting that circEGFR can accelerate EMT in TNBC cells [68]. Mechanistically, circEGFR facilitated the malignant advancement of TNBC through increasing the expression of EGFR via adsorbing miR-1299 [68]. CircBCBM1, generated from an lncRNA region within the FIRRE locus located on chromosome Xq26.2, was verified to be upregulated in BC brain metastasis cells, 231-BR, as well as tumor tissue and plasma specimens [69]. In vitro experiments substantiated that circBCBM1 accelerated 231-BR cell proliferation and migration. Likewise, in vivo experiments showed that circBCBM1 enhanced BC tumor growth and brain metastasis [69]. Mechanistically, circBCBM1 exhibited oncogenic effects in BC through sponging miR-125a and upregulating BRD4, leading to further enhancement of MMP9 expression via the SHH signaling pathway [69]. Intriguingly, MMP9 could induce BC brain metastasis via facilitating cells’ trans-endothelial migration and blood–brain barrier permeability [69]. Upregulated circ-TRIO in TNBC cell lines was validated using qRT-PCR detection, and the overexpression of circ-TRIO had a positive correlation with the degree of malignancy of BC cells [70]. Subsequent studies demonstrated that circ-TRIO knockdown mitigated the proliferation, invasion and migration of TNBC cells, while circ-TRIO overexpression led to the opposite impacts [70]. Furthermore, TNBC xenograft models were established to observe that tumor volume and weight were augmented in the circ-TRIO group compared with that in the control group, and the circ-TRIO group had more lung metastatic nodules, indicating that circ-TRIO strengthened cell proliferation and metastasis in TNBC in vivo [70]. Mechanistically, circ-TRIO sponged miR-432-5p to annul the suppressive impact of miR-432-5p on its downstream target CCDC58, thus boosting the expression of CCDC58 [70].

#### 4.2.2. Tumor Suppressors

CircKDM4B, also known as hsa_circ_0002926, exhibited limited expression in BC tissues, along with MDA-MB-468 and MDA-MB-231 cells [71]. Additionally, circKDM4B overexpression hindered cell migration and invasion in BC in vitro, whereas circKDM4B depletion caused the opposite effects [71]. Moreover, according to the findings from transwell and tube formation experiments, circKDM4B impeded the migration and tube-formation capability of human umbilical vein endothelial cells (HUVECs), whereas circKDM4B depletion facilitated the migration and tube formation capability of HUVECs [71]. Similarly, by performing IHC with anti-CD34 antibody, Guo et al. observed that the LV5-circKDM4B group had lower microvessel density than the LV5-NC group, indicating that circKDM4B suppressed angiogenesis in vivo [71]. Further in vivo experiments confirmed that circKDM4B could repress tumor growth and metastasis in nude mice [71]. Mechanistically, circKDM4B repressed BC progression by adsorbing miR-675 and subsequently elevating the expression of NEDD4L, which contributed to catalyzing ubiquitination of PI3KCA, thus restricting PI3K/AKT signaling and the secretion of VEGFA [71]. CircNR3C2 (hsa_circ_0071127), generated from back-splicing of the exon2 of NR3C2 gene, was markedly underexpressed in TNBC, and inversely associated with the distant metastasis and lethality of invasive BC [72]. CCK-8 and colony formation assays illustrated that overexpressing circNR3C2 in BC impeded cell proliferation and reproductive ability. In vivo tumor formation and metastasis assays showed that circNR3C2 overexpression suppressed tumor formation and reduced lung metastasis as well as metastatic pulmonary nodules [72]. Furthermore, circNR3C2 was demonstrated to hinder tumor migration and invasion using transwell and wound healing assays [72]. Mechanism studies indicated that circNR3C2 exerted suppressive effects on BC progression through serving as a miR-513a-3p sponge to enhance HRD1 expression, resulting in polyubiquitination-mediated degradation of Vimentin via proteasome [72]. Circ_ATAD3B was significantly decreased in BC tumor tissues, as evidenced by GEO datasets and qRT-PCR [73]. Circ_ATAD3B can function as a sponge for miR-570-3p and restrain cell survival and proliferation through increasing the expression of its downstream target MX2 [73]. Interestingly, the inhibitory impact of circ_ATAD3B on BC cell malignant phenotype was rescue by upregulation of miR-570-3p and downregulation of MX2 [73].

CircNOL10, derived from exons 6-12 of the NOL10 gene through back-splicing, also termed hsa_circ_0000977, exhibited decreased expression in BC tissues and cells [74]. A CCK-8 assay substantiated that upregulation of circNOL10 suppressed the viability of MDA-MB-231 and BT-549 cells, while circNOL10 depletion augmented the viability of MDA-MB-468 cells [74]. Plate clonality assay revealed that circNOL10 upregulation caused a remarkable reduction in the rate of colony formation in MDA-MB-231 and BT-549 cells, while depletion of circNOL10 resulted in an elevated rate of colony formation in MDA-MB-468 cells [74]. As presented by flow cytometry analysis, circNOL10 upregulation led to cell cycle arrest, while circNOL10 depletion facilitated cell cycle progression. A Transwell experiment elucidated that enhanced expression of circNOL10 caused an obvious reduction in cell migration and invasion, whereas circNOL10 depletion resulted in elevated cell metastasis [74]. Notably, Western blot analysis revealed that circNOL10 overexpression led to an increase in E-cadherin expression and a decrease in Vimentin and N-cadherin expression. Conversely, depletion of circNOL10 had the opposite effect on the expression of these markers associated with EMT [74]. Furthermore, in vivo experiments proved that circNOL10 can decelerate the growth of xenograft tumors in nude mice. Mechanistically, circNOL10 exerted suppressive effects on BC advancement by facilitating SOCS2 expression and deactivating JAK2/STAT5 signaling through absorption of miR-767-5p [74]. The expression of circDUSP1 was unveiled to be diminished in TNBC tissues and cells [75]. In-depth studies demonstrated that overexpressing circDUSP1 dramatically encumbered TNBC cell proliferation, migration, invasion and EMT, and attenuated tumor growth and metastasis in nude mice [75]. Mechanism analysis disclosed that circDUSP1 could facilitate the expression of DACT2 through acting as an endogenous miR-761 sponge, ultimately suppressing TNBC development [75].

Low expression of circPAPD4 was present in BC tissues and cells according to qRT-PCR results [76]. CircPAPD4 overexpression could substantially repress cell proliferation and induce cell apoptosis in BC in vitro and remarkably restrain xenograft tumor growth in nude mice in vivo [76]. In terms of mechanism, circPAPD4 repressed proliferation and accelerated apoptosis in BC through boosting CREBZF expression via competitively binding to miR-1269a [76]. Intriguingly, CREBZF could inhibit STAT3 dimerization and ADAR1 expression, further promoting circPAPD4 expression [76]. CircSLC8A1 was notably downregulated in BC tissues and cell lines, especially in MCF7 and T47D cells, and the expression of circSLC8A1 had negative correlation with clinical severity and unfavorable prognosis [77]. CCK-8 and colony formation experiments illustrated that circSLC8A1 upregulation markedly retarded MCF7 and T47D cell proliferation and colony formation capabilities, which was further validated by EdU incorporation assay [77]. Additionally, it was verified that the overexpression of circSLC8A1 decreased the migratory and invasive abilities of MCF7 and T47D cells as demonstrated by Transwell migration and invasion assays [77]. Furthermore, the results of xenograft models showed that upregulation of circSLC8A1 resulted in a palpable decrease in tumor size in MCF7 and T47D groups, suggesting that circSLC8A1 can suppress tumor growth in vivo [77]. Mechanistically, circSLC8A1 could adsorb miR-671 to enhance the expression of KLF16, a transcriptional activator of PTEN, thereby upregulating PTEN and subsequently inactivating PI3k/Akt signaling, ultimately inhibiting BC tumorigenesis [77]. Low circRNA_000554 expression was identified in BC tissues and cells, and circRNA_000554 was mostly localized in cytoplasm [78]. Overexpression of circRNA_000554 in BC cells hindered EMT, cell migration and invasion; induced cell cycle arrest in the G0/G1 phase; and facilitated cell apoptosis and autophagy [78]. In addition, in a xenograft tumor model assay, circRNA_000554 upregulation dramatically diminished tumor volume and weight in nude mice [78]. Mechanistically, circRNA_000554 suppressed BC progression through upregulating ZFP36 by adsorbing miR-182 [78].

## 5. CircRNAs as Diagnostic and Prognostic Biomarkers in Breast Cancer

A lack of reliable diagnostic and prognostic biomarkers results in the progression of BC to a stage where surgical resection is no longer feasible, which has become a pivotal issue in the field of BC research. Owing to their resistance to RNA enzyme-mediated degradation, circRNAs could be detected in diverse body fluids, such as blood, saliva and urine [83]. This non-invasive peculiarity makes circRNAs attractive as promising markers for BC screening and monitoring.

### 5.1. CircRNAs within BC Cells

CircCDYL, upregulated in the serum and tumor tissues of BC patients, was reported to be positively correlated with tumor burden and negatively correlated with OS and clinical response to treatment, indicating that circCDYL could be a promising molecule for forecasting the treatment response and prognosis of BC patients [65]. Hsa_circ_0067842, highly expressed in BC tissues and cells, was remarkably related to short disease-free survival (DFS) and OS of patients with BC [49]. Multivariate analysis results indicated that hsa_circ_0067842 can act as an independent prognostic factor for BC patients (*p* = 0.011). Collectively, hsa_circ_0067842 can be exploited as a promising prognostic biomarker in BC [49]. CircDNAJC11, overexpressed in BC tissues and cells, was unveiled to be linked to TNM stage according to the clinicopathological characteristics of BC patients [63]. The receiver operating characteristic (ROC) curve revealed that circDNAJC11 had considerable diagnostic value in BC. Additionally, patients with higher levels of circDNAJC11 had a markedly diminished OS rate in comparison to those with lower circDNAJC11 expression, implying that circDNAJC11 overexpression was associated with unfavorable prognosis of patients with BC [63]. Moreover, multivariate cox regression models demonstrated that high levels of circDNAJC11 expression may be an independent risk factor influencing the prognosis of BC patients [63]. CircEGFR was upregulated in TNBC tissues and cell lines, and increased expression of circEGFR was revealed to correlate with poor OS and DFS of TNBC patients, indicating that circEGFR could be a potential biomarker for TNBC diagnosis and prognosis [68]. Nevertheless, circEGFR expression levels had no notable correlation with age, TNM stage, tumor volume, lymphatic metastasis or vascular invasion [68].

The expression of circWSB1 appeared to be elevated in BC tissues and was linked to the T stage of BC patients [33]. According to the Kaplan–Meier survival analysis, patients exhibiting elevated circWSB1 levels had worse OS than those with lower circWSB1 levels [33]. Additionally, the application of recurrence analysis demonstrated a positive association between circWSB1 levels and the recurrence rate among patients with BC [33]. Moreover, as demonstrated by multivariate analysis, circWSB1 may serve as a standalone risk factor for BC patients. Collectively, circWSB1 could be a prognostic marker for BC patients [33]. Wang et al. found that the expression of circ-TRIO was related to recurrence in BC patients [70]. Additionally, these investigators discovered that circ-TRIO expression was markedly related to both DFS and OS in patients with TNBC, and higher circ-TRIO expression contributed to a poorer prognosis [70]. Further univariate and multivariate analyses of both OS and DFS elucidated that circ-TRIO expression might be an independent prognostic predictor for TNBC [70]. CircNOL10 was lowly expressed in BC tissues and cells, and low circNOL10 expression had intimate correlation with advanced TNM stage, larger tumor volume, lymphatic metastasis and dismal prognosis, but had no association with PR status, ER status, HER-2 status, age or menopause [74]. In addition, Kaplan–Meier survival analysis manifested that patients with circNOL10 downregulation had shorter OS [74]. Collectively, circNOL10 can be exploited as a potential prognostic factor for BC patients. In BC tissues, circPAPD4 expression levels were reduced, and this reduction was intimately related to advanced TNM stage, larger tumor size as well as higher Ki-67 expression [76]. Moreover, in comparison with patients with higher circPAPD4 expression, those with lower circPAPD4 expression displayed an unfavorable recurrence-free survival (RFS) [76]. Furthermore, Cox multivariable regression analysis revealed that circPAPD4 expression might be utilized as an independent prognostic biomarker for RFS in BC [76].

### 5.2. Exosomal circRNAs

Exosomes, a type of extracellular vesicle (EV) with a diameter of ~40–100 nm, are secreted by various cell types [84]. Exosomes containing different kinds of proteins, nucleic acids and lipids can mediate intercellular communication through releasing their cargo into the recipient cells, thereby regulating various pathophysiological processes [85]. For instance, high-dose human umbilical cord blood endothelial progenitor cell-derived EVs (EPC-EVs) can boost the regenerative functions of EPCs without changing their endothelial characteristics [86]. Human placental exosomes are involved in induction of maternal systemic immune tolerance through reprogramming circulating monocytes [87]. Exosomal delivery of 7SK lncRNA led to decreased viability, proliferation, tumorigenicity and aggressiveness in TNBC cells [88]. Recently, increasing attention has focused on the role of exosomal circRNAs as non-invasive biomarkers for BC diagnosis and prognosis.

Exosomal circRNA cSERPINE2 was substantially overexpressed in BC tissues relative to paired adjacent tissues, and BC patients with larger tumor volume and lymphatic metastasis showed higher cSERPINE2 expression [79]. Additionally, Kaplan–Meier survival analysis illustrated that higher cSERPINE2 expression in BC patients was related to shorter OS and RFS, predicting unfavorable prognosis [79]. Cox proportional hazards regression models indicated that increased cSERPINE2 expression can be regarded as an independent prognostic indicator for OS along with RFS in BC patients [79]. In terms of mechanism, tumor exosomal cSERPINE2 released into the tumor immune microenvironment (TIME) was internalized by tumor associated macrophages (TAMs) and induced the secretion of IL-6 in TAMs through sponging miR-513a-5p to upregulate MALT1 expression to activate the NF-κB pathway, ultimately boosting BC cell proliferation and invasion [79]. Notably, IL-6 can elevate the levels of EIF4A3 and CCL2 in tumor cells via activation of the JAK2/STAT3 pathway, further facilitating tumor cSERPINE2 biogenesis and inducing TAMs recruitment [79]. CircRHOT1 expression was evidently augmented in exosomes derived from BC cells and the serum of BC patients compared with the corresponding control groups [80]. In addition, ROC analysis was applied to assess the diagnostic potential of exosomal circRHOT1 in BC and found that the area under the curve (AUC) for exosomal circRHOT1 was 0.8300 (*p* < 0.01), further validating that exosomal circRHOT1 may bear potential as a diagnostic biomarker for BC [80]. Furthermore, circRHOT1 sponged miR-204-5p and thus elevated the expression of PRMT5, which accelerated BC cell proliferation, EMT, invasion and migration and encumbered apoptosis [80].

**Figure 2:**
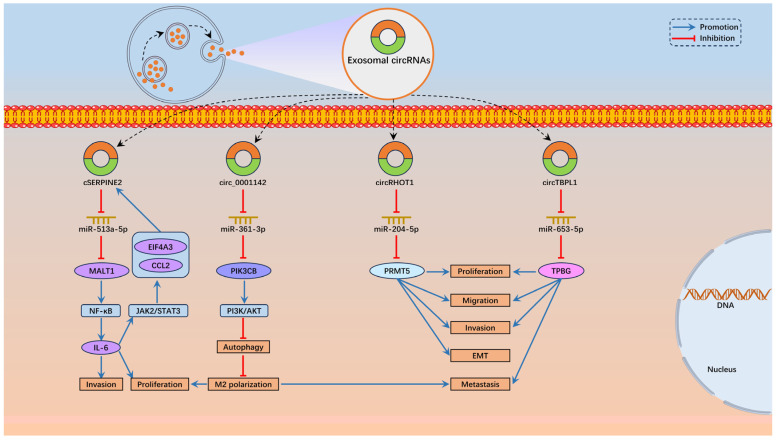
Exosomal circRNAs in BC.

CircTBPL1, generated from five exons of TBPL1 gene through back-splicing, was discovered to be distinctly enriched in exosomes derived from cancer-associated fibroblasts (CAFs) [81]. When CAF-derived exosomal circTBPL1 was transferred to BC cells, cell proliferation, invasion, migration and metastasis were remarkably facilitated [81]. Mechanistically, exosomal circTBPL1 exhibited tumor-promoting effects in BC through adsorbing miR-653-5p and elevating TPBG expression. Therefore, exosomal circTBPL1 might become a marker for BC diagnosis [81]. Highly expressed circ_0001142 was present in BC tissues and cells, and circ_0001142 upregulation was correlated with advanced TNM stage, lymph node metastasis and unfavorable prognosis [82]. Interestingly, endoplasmic reticulum stress induced the secretion of tumor exosomes enriched with circ_0001142, which inhibited autophagy and strengthened M2 polarization of macrophages to boost tumor proliferation and metastasis in BC through sponging miR-361-3p to enhance PIK3CB expression to induce the activation of PI3K/AKT pathway [82]. Taken together, the results mentioned above may enable the selection of circ_0001142 as a latent biomarker for BC diagnosis and prognosis. Figure 2 depicts how exosomal circRNAs participate in regulating BC progression.

## 6. CircRNAs as Therapeutic Targets in Breast Cancer

Currently, the growing emergency of drug resistance in the treatment of BC has become a thorny issue that remains to be addressed [89]. Studies have demonstrated that several circRNAs are tightly linked to BC drug resistance (Table 3). For instance, one group found that circEGFR upregulation markedly enhanced pirarubicin (THP) resistance and elevated the half-maximal inhibitory concentration (IC_50_) in MDA-MB-231 TNBC cells, whereas circEGFR depletion significantly facilitated THP treatment efficacy and diminished the IC_50_ in MDA-MB-231 cells [68]. Subsequent studies demonstrated that THP remarkably retarded cell proliferation, invasion, migration as well as EMT in MDA-MB-231 cells through downregulation of circEGFR. Therefore, circEGFR may be a promising target for TNBC therapy [68]. Ling et al. unveiled that circCDYL2, circSAT1 and circSPECC1L showed significant upregulation in trastuzumab-resistant BC patients [90]. Subsequently, circCDYL2 expression was confirmed to be evidently increased in trastuzumab-resistant BC cell lines, as well as HER2-positive BC tissues [90]. Notably, HER2^+^ BC patients with circCDYL2 overexpression relapsed rapidly and displayed shorter DFS and OS after anti-HER2 treatment in comparison with those with low circCDYL2 expression [90]. CircCDYL2 could stabilize GRB7 via inhibiting its ubiquitination degradation and strengthen the interaction between GRB7 and FAK, thus sustaining the activities of AKT and ERK1/2 and further promoting trastuzumab resistance in BC [90]. Circ-BGN expression was remarkably elevated in trastuzumab-resistant BC cells and tissues, which was linked to unfavorable OS [91]. Importantly, circ-BGN knockdown subdued BC cell viability and restored the sensitivity of HER2-positive BC cells to trastuzumab [91]. Mechanism research expounded that circ-BGN directly bound to OTUB1 and SLC7A11 and boosted SLC7A11 deubiquitination mediated by OTUB1, thus suppressing ferroptosis and further enhancing trastuzumab resistance in HER2-positive BC [91]. Collectively, targeting circ-BGN/OTUB1/SLC7A11 may contribute to reducing trastuzumab resistance in HER2-positive BC patients.

Wang et al. used a qRT-PCR assay to validate 10 significantly upregulated circRNAs in trastuzumab-resistant HER2-positive BC cells, of which circ-β-TrCP exhibited the largest upregulation proportion [92]. In addition, patients with higher circ-β-TrCP expression had shorter OS than those with lower circ-β-TrCP expression [92]. Subsequently, circ-β-TrCP was demonstrated to confer trastuzumab resistance through modulating NRF2-mediated antioxidant pathway, which was independent of the KEAP1 pathway [92]. More specifically, β-TrCP-343aa, a novel peptide encoded by circ-β-TrCP, could competitively bind to NRF2 and hinder SCF^β-TrCP^-mediated proteasomal degradation of NRF2 in a GSK3 activity-dependent manner, thus transcriptionally upregulating several antioxidant genes and promoting trastuzumab resistance [92]. Interestingly, NRF2 could transcriptionally suppress eIF3j to block the inhibitory effect of eIF3j on the translation ability of circ-β-TrCP, leading to the establishment of a positive feedback circuit between NRF2 and β-TrCP-343aa, hastening the development of trastuzumab resistance [92]. The presence of elevated levels of circFAT1 was detected in oxaliplatin-resistant BC tissues and cells. Subsequent functional experiments revealed that circFAT1 depletion caused a decline in the expression of genes associated with chemoresistance [93]. Additionally, circFAT1 silencing dramatically diminished IC_50_ value of oxaliplatin, invasion and migration, and induced apoptosis in BC cells resistant to oxaliplatin [93]. Further mechanism research revealed that circFAT1 can strengthen SKA1 expression by sponging miR-525-5p, thus enabling the activation of the Notch and Wnt pathway, facilitating oxaliplatin resistance in BC [93].

CircPVT1, which showed overexpression in ERα-positive BC cells and tumor samples, was functionally indispensable for facilitating ERα-positive breast tumorigenesis and tamoxifen resistance [94]. CircPVT1 stabilized ESR1 expression, activated estrogen/ERα-target genes via adsorbing miR-181a-2-3p, and suppressed the type I IFN signaling pathway along with anti-tumor immunity through interacting with MAVS protein and disrupting the generation of RIGI-MAVS complex, thereby boosting ERα-positive BC cell growth and tamoxifen resistance [94]. However, depletion of circPVT1 was demonstrated to restrain BC cell growth and resensitize tamoxifen-resistant MCF7 cells to tamoxifen therapy, suggesting that circPVT1 could act as a promising target for ERα-positive BC therapy clinically [94]. By reanalyzing two public datasets from GEO, Li et al. revealed nine circRNAs that showed overexpression in BC tissues and cells resistant to tamoxifen [95]. Subsequently, these investigators determined that circRNA-SFMBT2, derived from exons 5–8 of SFMBT2 gene, exhibited significantly higher expression in ER^+^ BC cells than in ER^−^ cells. They also uncovered a connection between circRNA-SFMBT2 overexpression and both larger tumor size and dismal prognosis in ER^+^ BC patients [95]. Additionally, overexpression of circRNA-SFMBT2 in ER^+^ BC led to an increase in cell growth and tamoxifen resistance [95]. Mechanistically, relying on its distinctive tertiary structure, circRNA-SFMBT2 could bind to the DBD and AF2 regions of ERα to recruit RNF181 to the AF1 region of ERα. Moreover, the circRNA-SFMBT2/RNF181 axis strengthened ERα stability by differentially modulating K-63-linked and K48-linked ERα ubiquitination, leading to overexpression of ERα target genes as well as BC progression [95]. Notably, depletion of circRNA-SFMBT2 drastically hindered cell proliferation along with tamoxifen resistance in BC [95]. Hence, targeting circRNA-SFMBT2 may constitute an alternative strategy for overcoming tamoxifen resistance and repressing BC progression. Higher expression of circTRIM28 was observed in tamoxifen-resistant BC tissues and MCF7/R and MDAMB-231/R cells, and the increased circTRIM28 levels were correlated with decreased post-operative survival in BC patients [96]. Knockdown of circTRIM28 resulted in strengthened tamoxifen sensitivity and cell apoptosis, and also hindered cell development in BC cells [96]. Mechanistically, circTRIM28 induced tamoxifen resistance and tumor growth in BC through increasing HMGA2 expression via sponging miR-409-3p [96].

The expression of circWAC was dramatically upregulated in TNBC tissues and was related to worse prognosis in patients with TNBC [97]. Further experiments confirmed that circWAC upregulation induced paclitaxel (PTX) resistance in TNBC cells, whereas circWAC downregulation augmented the sensitivity of TNBC cells to PTX, indicating the potential of circWAC as a therapeutic target in TNBC [97]. In terms of mechanism, circWAC adsorbed miR-142 to nullify the inhibitory impact of miR-142 on WWP1, thus boosting WWP1 levels and subsequently activating PI3K/AKT pathway, eventually reinforcing PTX resistance in TNBC [97]. The lapatinib-resistant BC tissues and cells exhibited an increased expression of circ-MMP11 [98]. Notably, depletion of circ-MMP11 led to enhanced lapatinib sensitivity in lapatinib-resistant BC cells through attenuating cell proliferation, migration and invasion [98]. Mechanistically, circ-MMP11 facilitated lapatinib resistance in BC cells by facilitating ANLN expression through functioning as a miR-153-3p sponge [98]. CircUBAP2 expression was notably upregulated in cisplatin-resistant TNBC tissues and cells, as validified by qRT-PCR detection [99]. Additionally, the sensitivity of TNBC cells to cisplatin was reduced after knockdown of circUBAP2 [99]. CircUBAP2 could function as a sponge for miR-300 and elevate the expression of ASF1B, which subsequently activated PI3K/AKT/mTOR (PAM) pathway, expediting resistance of TNBC to cisplatin, providing a promising therapeutic target for TNBC patients with cisplatin resistance [99]. Circ_0001667 was remarkably upregulated in Adriamycin (ADM)-resistant BC tissues, as well as MCF-7/ADM and MDA-MB-231/ADM cells [100]. Furthermore, circ_0001667 depletion was elucidated to mitigate ADM resistance, cell proliferation, migration and invasion in MCF-7/ADM and MDA-MB-231/ADM cells [100]. Subsequent experiments demonstrated that circ_0001667 boosted ADM resistance and BC progression through sponging miR-4458 and enhancing the expression of NCOA3 [100]. Therefore, targeting circ_0001667 may be conducive to attenuating ADM resistance and restraining tumor progression in BC.

## 7. Conclusions and Perspectives

Breast cancer is a unique and devastating disease that poses a substantial threat to women’s health. Given the widespread prevalence of BC across the world and its potential to result in significant morbidity and mortality, conducting in-depth research on BC is of immense significance. In this article, we outlined how circRNAs are engaged in regulation of cellular processes in BC, such as proliferation, invasion, migration, apoptosis, angiogenesis, EMT and metastasis. Most of the differentially expressed circRNAs discussed in this article regulate BC progression via serving as miRNA sponges. However, further exploration is warranted to understand how circRNAs exert their impacts on BC progression through other mechanisms such as affecting parental gene expression, encoding proteins or peptides, interacting with proteins, and so on. It has been confirmed that the expression levels of circRNAs could be regarded as both diagnostic and prognostic indicators for BC. However, there is scant knowledge regarding the roles of exosomal circRNAs as diagnostic and prognostic markers for BC. Consequently, dedicating efforts towards further investigation of exosomal circRNAs as markers for BC diagnosis and prognosis is of paramount importance. Furthermore, the potential of circRNAs as therapeutic targets will undoubtedly offer a new alternative treatment approach for BC. Nevertheless, delivering circRNAs to specific regions of the human body and avoiding the occurrence of potential immune rejection may pose evident hurdles that must be addressed urgently in developing effective BC treatment options in the future.

## Figures and Tables

**Table 2:** Expression profiles of circRNAs in BC.

Experimental Model	Method	Differentially Expressed circRNAs	Reference
BC tissues	RNA sequencing	70 circRNAs were upregulated and 78 circRNAs were downregulated.	[48]
BC tissues	CircRNA microarray analysis	89 downregulated and 129 upregulated circRNAs were reported.	[49]
BC tissues	CircRNA high-throughput sequencing	136 increased circRNAs were found.	[50]
BC tissues	CircRNA microarray analysis	256 upregulated circRNAs and 277 downregulated circRNAs were identified.	[51]
BC tissues	ceRNA microarray probes	2375 and 1995 circRNAs were increased and decreased, respectively.	[52]
ER-positive BC tissues	CircRNA microarray analysis	1700 circRNAs were downregulated and 1953 circRNAs were upregulated.	[53]
BC cell lines	High-throughput RNA sequencing	16 downregulated and 7 upregulated circRNAs were discovered.	[54]
BCBM cell lines	RNA-sequencing	191 circRNAs were decreased and 215 circRNAs were elevated.	[55]

**Table 3:** CircRNAs involved in drug resistance in BC.

Therapeutic Drugs	CircRNAs	Expression	Effect on Drug Resistance	Pathways	Reference
Pirarubicin	circEGFR	Up	Promoting	circEGFR/miR-1299/EGFR	[68]
Trastuzumab	circCDYL2	Up	Promoting	circCDYL2/GRB7/FAK/AKT and ERK1/2	[90]
Trastuzumab	circ-BGN	Up	Promoting	circBGN/OTUB1/SLC7A11	[91]
Trastuzumab	circ-β-TrCP	Up	Promoting	circ-β-TrCP/β-TrCP-343aa/NRF2	[92]
Oxaliplatin	circFAT1	Up	Promoting	circFAT1/miR-525-5p/SKA1/Notch and Wnt pathway	[93]
Tamoxifen	circPVT1	Up	Promoting	circPVT1/miR-181a-2-3p/ESR1; circPVT1/MAVS	[94]
Tamoxifen	circRNA-SFMBT2	Up	Promoting	circRNA-SFMBT2/RNF181	[95]
Tamoxifen	circTRIM28	Up	Promoting	circTRIM28/miR-409-3p/HMGA2	[96]
Paclitaxel	circWAC	Up	Promoting	circWAC/miR-142/WWP1	[97]
Lapatinib	circ-MMP11	Up	Promoting	circ-MMP11/miR-153-3p/ANLN	[98]
Cisplatin	circUBAP2	Up	Promoting	circUBAP2/miR-300/ASF1B/PI3K/AKT/mTOR	[99]
Adriamycin	circ_0001667	Up	Promoting	circ_0001667/miR-4458/NCOA3	[100]

## Abbreviations

circRNA: circular RNA; BC: breast cancer; TNBC: triple-negative breast cancer; PTC: papillary thyroid cancer; pre-mRNA: precursor mRNA; EcircRNA: exonic circRNA; ciRNA: intronic circRNA; EIcircRNA: exon–intron circRNA; RBP: RNA-binding protein; ssRNA: single-stranded RNA; QKI: Quaking; ICC: intrahepatic cholangiocarcinoma; MEK1: mitogen-activated protein kinase 1; ERK2: mitogen-activated protein kinase 2; STAT3: signal transducer and activator of transcription 3; hnRNPM: heterogeneous nuclear ribonucleoprotein M; PRDX2: peroxiredoxin 2; PARP1: poly ADP-ribose polymerase 1; HSPB1: heat shock protein beta 1; AUF1: AU-rich element (ARE) RNA-binding factor 1; ORF: open reading frame; HCC: hepatocellular carcinoma; DE: differentially expressed; qRT-PCR: quantitative real-time polymerase chain reaction; EMT: epithelial–mesenchymal transition; BCBM: breast cancer brain metastasis; KLF5: Krüppel-like factor 5; FUS: RNA-binding protein FUS; CXCR4: C-X-C chemokine receptor type 4; EIF4A3: eukaryotic initiation factor 4A-III; SOS1: son of sevenless 1; CCK-8: cell counting kit-8; EdU: 5-ethynyl-2′-deoxyuridine; FASN: fatty acid synthase; EIF4A3: eukaryotic translation initiation factor 4A3; TCF4: transcription factor 4; PDK1: 3-phosphoinositide dependent protein kinase-1; OS: overall survival; DFS: disease-free survival; HK2: hexokinase 2; IGF2BP1: insulin-like growth factor 2 mRNA-binding protein 1; IC_50_: half-maximal inhibitory concentration; THP: pirarubicin; BRD4: bromodomain-containing 4; SHH: Sonic hedgehog; MMP9: matrix metallopeptidase 9; YAP1: yes-associated protein 1; CDT1: Cdc10-dependent transcript 1; CCDC58: coiled-coil domain containing 58; HUVECs: human umbilical vein endothelial cells; NEDD4L: NEDD4-like E3 ubiquitin protein ligase; HRD1: HMG-CoA reductase degradation protein 1; SOCS2: suppressors of cytokine signaling 2; PAPD4: PAP-associated domain-containing protein 4; CREBZF: CREB/ATF BZIP transcription factor; ADAR1: adenosine deaminase acting on RNA 1; RFS: recurrence-free survival; KLF16: Krüppel-like factor 16; PTEN: phosphatase and tensin homolog deleted from chromosome 10; IHC: immunohistochemistry; ZFP36: zinc finger protein 36; EV: extracellular vesicle; TIME: tumor immune microenvironment; TAMs: tumor-associated macrophages; IL-6: interleukin-6; EPC-EVs: endothelial progenitor cell-derived extracellular vesicles; PRMT5: protein arginine methyltransferase 5; ROC: receiver operating characteristic; AUC: area under the curve; CAFs: cancer-associated fibroblasts; TPBG: trophoblast glycoprotein; HER2: human epidermal growth factor receptor 2; GRB7: growth factor receptor-bound protein 7; FAK: focal adhesion kinase 1; IFN: interferon; MAVS: mitochondrial antiviral signaling; SLC7A11: solute carrier family 7, member 11; ANLN: anillin; HMGA2: high-mobility group AT-hook 2; SKA1: spindle and kinetochore-associated complex subunit 1; ASF1B: anti-silencing function 1B histone chaperone.
